# Connectivity-Based Parcellation of the Cortical Mantle Using *q*-Ball Diffusion Imaging

**DOI:** 10.1155/2008/368406

**Published:** 2008-03-26

**Authors:** Muriel Perrin, Yann Cointepas, Arnaud Cachia, Cyril Poupon, Bertrand Thirion, Denis Rivière, Pascal Cathier, Vincent El Kouby, André Constantinesco, Denis Le Bihan, Jean-François Mangin

**Affiliations:** ^1^NeuroSpin Institut d'Imagerie BioMédicale, Commissariat l'Energie Atomique (CEA), Gif-sur-Yvette 91191, France; ^2^Institut Fédératif de Recherche 49, Gif-sur-Yvette 91191, France; ^3^GE Healthcare, 11 avenue Morane Saulnier, Vélizy 78457, France; ^4^Inserm U.797, CEA-INSERM Research Unit “Neuroimaging & Psychiatry”, Service Hospitalier Frédéric Joliot, Orsay, Orsay Cedex 91401, France; ^5^Parietal Project, INRIA Futurs, NeuroSpin, Gif-sur-Yvette 91191, France; ^6^Service de Biophysique et Médecine Nucléaire, Hopital de Hautepierre, 1 ave Molière, Strasbourg 6708, France

## Abstract

This paper exploits the idea that each individual brain region has a specific connection profile to create parcellations of the cortical mantle using MR diffusion imaging. The parcellation is performed in two steps. First, the cortical mantle is split at a macroscopic level into 36 large gyri using a sulcus recognition system. Then, for each voxel of the cortex, a connection profile is computed using a probabilistic tractography framework. The tractography is performed from *q* fields using regularized particle trajectories. Fiber ODF are inferred from the *q*-balls using a sharpening process focusing the weight around the *q*-ball local maxima. A sophisticated mask of propagation computed from a T1-weighted image perfectly aligned with the diffusion data prevents the particles from crossing the cortical folds. During propagation, the particles father child particles in order to improve the sampling of the long fascicles. For each voxel, intersection of the particle trajectories with the gyri lead to a connectivity profile made up of only 36 connection strengths. These profiles are clustered on a gyrus by gyrus basis using a *K*-means approach including spatial regularization. The reproducibility of the results is studied for three subjects using spatial normalization.

## 1. INTRODUCTION

Diffusion magnetic resonance imaging is a probe allowing noninvasive studies of the microscopic structure of brain tissues. For instance, inside white matter, preferential orientations of fiber bundle axonal membranes induce anisotropy of the local Brownian motion of water molecules. The fiber orientation can be inferred from this anisotropy. Hence, one of the most attractive applications of diffusion imaging is the tractography of white matter fiber bundles and the inference of brain connectivity.

Tractography has been developped first from diffusion tensor imaging (DTI) [1], a technique indicating for each voxel the direction of the highest amplitude of the diffusion process. Assuming that this direction corresponds to the main fiber orientation inside the voxel, some of the tracts can be reconstructed step by step [2–4]. Unfortunately this simplistic approach can not resolve fiber crossings, which are numerous in the brain. The problem is partially overcome with either preprocessing of tensor field [3, 5–9] or more sophisticated methods of tractography involving either regularization of the bundle trajectories [10, 11] or probabilistic strategies based on Monte Carlo sampling and models of uncertainty about fiber orientations [12–14]. The fiber orientation distribution function (ODF) inferred in each voxel from DTI, however, is not sufficient to map successfully the large-scale connectivity of the cortex because of the amount of crossings involved [15].

The emergence of high angular resolution diffusion imaging (HARDI) provides the opportunity to better model water mobility in fiber crossing. Hence, more reliable mapping of the corticocortical pathways can be achieved, which is exploited in this paper. There is no consensus yet on the best way to interpret HARDI data for tractography [16–23]. The main issue is the choice of the method used to build fiber ODFs. In this paper, we explore the potential of *q*-ball imaging, a method pushing further than DTI the idea that the fiber directions can be inferred from the local maxima of the amplitude of water molecule radial displacements [24, 25].

The network of anatomical connections linking the neuronal elements of the human brain is still largely unknown [26, 27]. Therefore, compiling the connection matrix or the “connectome” of the human brain represents an indispensable foundation for basic and applied neurobiological research [27]. One of the challenges faced by this research program is that the structural elements of the human brain, in terms of interesting nodes for the connection matrix, are difficult to define. Attempting to assemble the human connectome at the level of single neurons is unrealistic and will remain infeasible at least in the near future. Nevertheless, a higher scale of representation is more attractive: there is an overwhelming evidence that human cognitive functions depend on the activity of large populations of neurons in distributed network. Unfortunately, brain areas and neuronal populations are difficult to delineate.

No single universally accepted parcellation scheme currently exists for the human brain. In the cerebral cortex, neurons are arranged in an unknown number of anatomically distinct regions and areas, perhaps on the order of 100 or more [28]. The most standard parcellation, which has been proposed by Brodmann one hundred years ago from cytoarchitectonic criterions, cannot be mapped in vivo. Anyway, while cyto- and myeloarchitectonics are powerful methods to highlight anatomical segregation, animal studies have shown that further parcellations of architectonically homogeneous areas can be obtained using connectivity [29]. Therefore, the most promising avenue for parcellating the brain and compiling the brain connectome originates from the notion that individual brain regions maintain individual connection profiles [27]. What defines a segregated brain region is that all its structural elements share highly similar long-range connectivity patterns, and that these patterns are dissimilar between regions. These connectivity patterns determine the region functional properties [30], and also allow their anatomical delineation and mapping.

Tractography has been used previously to distinguish thalamic areas using a lobar parcellation of the cerebral cortex as input [31]. For this application, each thalamus voxel was attached to the lobe with the strongest connection. The idea that the whole patterns of connectivity can be used to identify areal boundaries has been demonstrated in the human medial frontal cortex [32]. First, connection strengths from voxels within the medial frontal cortex to all other voxels in the rest of the brain were obtained. Connection profiles were then used to calculate a cross-correlation matrix, which was examined for the existence of distinct clusters of voxels with shared connection patterns. The resulting clusters matched an independent clustering of the same region obtained from functional imaging. The robustness of the approach has been studied further in [33]. Another successful local parcellation related to Broca's area has been recently achieved by another group in [34].

In this paper, we extend further the idea of parcellating the cerebral cortex using connectivity profiles. The main difference with the works mentioned above is that we address the parcellation of the complete cortical mantle. Following the approach of Oxford group [31–33], our purpose would involve the difficult clustering of a huge cross-correlation matrix. To overcome this problem, our parcellation framework relies on an initial macroscopic parcellation of the cortex into 36 large gyri performed with a pipeline of processing [35] provided in brainVISA framework (http://brainvisa.info). This initial parcellation is used to reduce each voxel connectivity profile to a short vector of 36 values, namely the strength of connection to each of the gyri. A second use of the gyral parcellation is to split the initial global clustering problem into 36 smaller problems: the gyri are clustered one by one. The justification leading to the use of a gyral parcellation to reduce the complexity of the problem lies in the strong link between this large scale division of the cortex and its functional and architectonic organizations [36, 37]. An additional argument stems from the hypothesis that the fiber bundle organization is deeply related to the folding patterns of the cerebral cortex [38].

In the following, we provide first a brief overview of our data, of the preprocessing steps and, of our choice for the fiber ODF. While this part of the paper is not detailed, it should be noted that we deal with especially high-quality datasets based on 200 directions of diffusion and a *b* value of 3000 s/mm^2^ increasing the contrast between crossing bundles. Moreover, a dedicated MR sequence and several steps of distortion correction are used to achieve a perfect alignment between the diffusion data and the high-resolution T1-weighted image used to compute the gyral parcellations. All this care is mandatory to address the mapping of the corticocortical interareal pathways. This dataset is provided to the community [39].

The next part describes the different steps of our parcellation method. We first describe our “probabilistic” tractography framework dedicated to *q*-ball fields and based on regularized particle trajectories. Our method includes several original refinements compared to its first introduction [40]: (1) an algorithm initially dedicated to the detection of the cortical sulci [41] is used to build a mask preventing the particles from spuriously crossing the cortical folds; (2) a local sharpening of the *q*-ball ODF is performed to concentrate the Monte Carlo sampling around the most probable fiber directions; (3) a processus creating children fibers during the tracking has been designed in order to improve the sampling of long bundles. The behavior of these refinements is illustrated using virtual phantoms of crossing computed via simulation of the random walks of the water molecules in a restricted geometric environment.

Then, we describe the method dedicated to the clustering of the voxels of the cortical mantle from the connectivity profile provided by the probabilistic tractography. We first rapidly recall the basic ideas underlying the computation of our gyral parcellation [35]. Then we describe the clustering algorithm, which is based on a *K*-means framework including a Markov random field regularization procedure. Finally, the method is tested with three brains through the clustering of 14 different gyri. We compare the connectivity matrices obtained for the 14 gyri and study the reproducibility of the clustering using a spatial normalization method.

## 2. METHOD

### 2.1. Fiber orientation distribution function

#### 2.1.1. Data acquisition

Diffusion-weighted and T_1_-weighted images were acquired in three healthy subjects on a GE Healthcare Signa 1.5 Tesla Excite II scanner provided with a 40 mT/m whole body gradient coil, eight receiver channel acquisition system, and an eight channel head surface coil.

 T_1_-weighted anatomical images were acquired using fast gradient echo with inversion recovery sequence (echo time 2 milliseconds, repetition time 9.9 milliseconds, inversion preparation time 600 milliseconds, flip angle 10°, bandwidth 12.5 kHz, FOV 24 cm, and repetition time 2 leading to a 15 minutes and 52 seconds scan time). Voxel size is 0.9375 × 0.9375 mm^2^ with a slice thickness of 1.2 mm. We used 2 repetitions in order to get a very good contrast to noise ratio simplifying grey/white classification.

Diffusion weighted data were acquired with HARDI scheme. The sequence is a dual spin echo (echo time is 93.2 milliseconds, volume repetition time is 19000 milliseconds, flip angle is 90°, bandwidth is 200 kHz, FOV is 24 cm). Voxel size is 1.98 × 1.98 mm^2^ with a slice thickness of 2 mm. The diffusion weighting was isotropically distributed along 200 directions using a *b* value of 3000 s/mm^2^. Along the acquisition, 25 additional volumes without diffusion sensitization were acquired and finally averaged to obtain a T2-weighted image perfectly aligned with the diffusion-weighted dataset. The scan time for this diffusion protocol was 72 minutes and 50 seconds. A phase map for diffusion data was acquired using a 2D double gradient echo sequence (echo time 4.5 milliseconds, slice repetition time 441 milliseconds, flip angle 60°, bandwidth 15 kHz, FOV 24 cm, and the same slice location as for diffusion protocol leading to a 3 minutes and 25 seconds scan time).

#### 2.1.2. Diffusion data preprocessing

**Figure 1:**
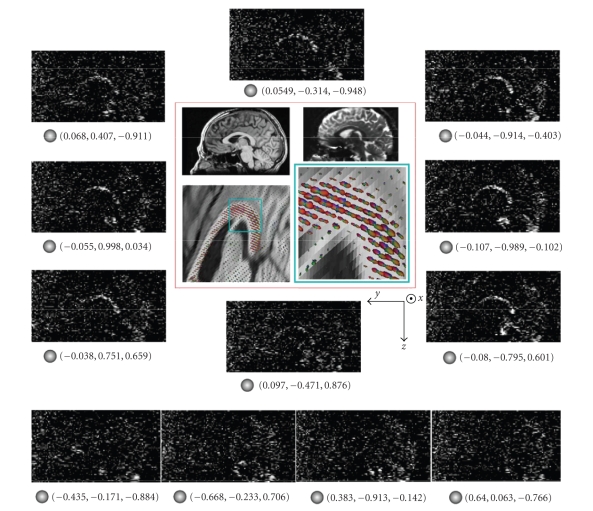
An illustration of the links between high *b* value diffusion-weighted data, Funk-Radon transform, diffusion ODF, and fiber ODF: the slice cuts the corpus callosum, whose fibers approximately follow the *X* direction. The figure proposes the aligned T1- and T2-weighted slices and a number of diffusion-weighted slices. When the diffusion gradient is not orthogonal to the fibers (unit vector under corresponding slice), most of the MR signal is destroyed by water diffusion: the corpus callosum is black (row of slices at the bottom). When the diffusion gradient is orthogonal to the fibers, water diffusion is restricted by the axonal membranes: some noisy signal survives in the corpus callosum. Hence, the sum of the raw signal along the equator around the *X* axis leads to a peak of the *q*-ball indicating the fiber direction. In this figure, the *q*-balls. are scaled according to their anisotropy.

The use of a twice refocusing diffusion module compensates for the echoplanar distortions, due to eddy currents, at the first order. However, two other kinds of geometric distortions had to be corrected, related to the nonlinearities of the gradients and to the local static field inhomogeneities induced by tissue/air interfaces. The first kind of distortions was overcome during reconstruction using GE warping procedures. The second kind related to susceptibility was corrected using the phase map [42, 43]. This method corrects intensity variation and voxel shifts caused by the local field inhomogeneities. After these procedures, T1 and diffusion-weighted datasets could be aligned perfectly using a rigid transformation. This transformation was computed by maximizing the mutual information between the T1-weighted image and the average T2-weighted image (cf. Figure 1).

#### 2.1.3. From *q* to fiber ODF

In order to develop a probabilistic tractography algorithm, HARDI datasets have to be converted into a fiber ODF in each voxel. Numerous ideas have been proposed for this purpose. The first class of approaches is based on models of the signal observed with one single bundle. The model is used to solve in each voxel a local inverse problem according to different alternative frameworks [16–21, 23, 44–46]. The HARDI signal is then explained as a mixture of models. These approaches convert HARDI data into a fiber ODF focusing on a small set of putative crossing fiber directions. Hence, the local distribution around each of these directions is an estimate of the uncertainty associated with the fiber orientation. It is beyond the scope of this paper to provide a comparison of all the existing approaches.

A second strategy consists in using iconic representations of the diffusion process to build the fiber ODF. This point of view does not rely on model fitting or deconvolution procedures. This is supposed to alleviate the risk of misinterpreting the MR data either because of some weaknesses of the model or because of some failure of the method used to perform the local inverse problem. Diffusion spectrum imaging (DSI), which provides for each voxel a 3D image of the water displacement probability distribution, is at the origin of this research direction [47]. DSI is based on sampling the 3D Fourier space of the water displacement distribution, which requires large pulsed field gradients. The radial projection of the diffusion function, called the diffusion ODF, is supposed to convey most of the information about the diffusion process required to guess fiber orientations [48]. It has been shown recently that the diffusion ODF can be approximated from HARDI acquisitions using a spherical tomographic inversion called the Funk-Radon transform, also known as the spherical Radon transform [24, 25]. In this paper, we use the ODF resulting from this numerical transform, which is called *q*-ball. The most recent refinement leading to a robust analytical reconstruction was not used here [22]. There is no consensus yet on the comparison between the diffusion ODF and the *q*-ball approximation for the accuracy of the fiber direction estimation [49, 50].

The exact link between *q*-ball and fiber ODF is still unclear, but simple hypotheses lead to convert the *q*-ball into interesting candidates for the fiber ODF. Assuming that at high *b* value most of the diffusion weight which stems from intra-axonal water leads to considering the amplitude of the radial displacement of water molecules a good marker of putative fiber directions. The simplest use of this idea consists in associating the *q*-ball local maxima with the fiber orientations, obtaining a small set of directions playing the same role as the sets provided by the model-based methods mentioned above. This approach assumes the equivalence between the local maxima of the *q*-ball and the local maxima of the fiber ODF, which can be discussed [24]. It is known to often fail when two fiber bundles cross with an angle of less than 45 degrees.

Pushing further the hypothesis of a strong equivalence between both ODFs, one can consider that the shape of the *q*-ball around a local maximum provides a good estimation of the uncertainty related to the orientation of the underlying fibers. This is the strategy chosen in this paper to sample the fiber directions during the probabilistic tractography. We will not address here the validity of this hypothesis, which requires a crucial research program based on physical phantoms of fiber crossing [51–53] and a better understanding of the physics of diffusion in biological tissues (choice of the *b* value, membrane permeability, number of compartments, etc.). This program, however, needs time to deliver some answers, which should not stop the development of tractography algorithms. These algorithms, indeed, have the possibility to use contextual knowledge, namely the neighborhood of a voxel, in order to tackle locally the inverse problem. Therefore, they can overcome some of the weaknesses of the current fiber ODFs and provide meaningful neuroscience results.

In the following, *q*-ball data are visualized according to the following rules. Each *q*-ball is represented by a spherical mesh. Each node of the mesh is moved outward according to the amplitude *ψ* of the water molecule displacement (more precisely the result of the radial summation of the diffusion function). In order to maximize the information provided by this deformation process, this move is computed as (*ψ* − min_*S*_(*ψ*))/(max_*S*_(*ψ*) − min_*S*_(*ψ*), where *S* is the sampled sphere of the current voxel. To improve visualization further, each node is given a color related to its orientation relative to the image axis: red for *x* axis (left-right), green for *y* axis (frontal-occipital), and blue for *z* axis (top-down), interpolated in between. Finally, the resulting mesh is sometimes scaled according to the *q*-ball anisotropy in order to highlight the regions with the largest influence on tractography (cf. Figure 1).

### 2.2. Probabilistic regularized tractography in *q*-ball fields

The probabilistic tractography framework used in this paper is an extension of a method described before [40]. This method has been designed in order to remain as simple as possible. It should be considered as a first attempt to explore the potential of the new generation of high-quality data recently provided by our MR physicists. Hence, this method aims at paving the way for more sophisticated developments [15]. The initial algorithm was based on regularized particle trajectories used to perform the Monte Carlo sampling of the white matter geometry. Validation has been provided using a crossing phantom made up of sheets of parallel haemodialysis fibers and through the successful tracking of the primary auditory tract in the human brain [40]. This last achievement was especially impressive because of the problematic crossing of this tract with a thick orthogonal pathway. However, the shortness of this tract and the simple geometry of the phantom were too favorable configurations to consider these first experiments as warrant of success with long cortico-cortical pathways. Therefore, in the following, after describing briefly the initial method (1, 2), three important refinements are proposed (3, 4, 5).

#### 2.2.1. Particles with inertia

Each origin voxel is spatially sampled in order to define the starting points of *n* particles. These particles move inside a continuous *q*-ball field defined by linear interpolation. The particles are given an initial speed in the direction corresponding to the maximum of the local *q*-ball. This *q*-ball is sampled in the 400 = 2∗200 directions of acquisitions.

Then, each particle moves with constant speed according to a simplistic sampling scheme: let us note *p*(*i*) the location of the particle at time *i*, and $\overset{\to }{v\left(i\right)}$ the direction of the particle speed at time *i*

(1) $$p(i+\delta t)=p(i)+\overset{\to }{v(i)}*\delta t.$$

**Figure 2:**
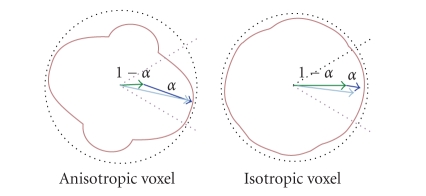
The normalized standard deviation of the *q*-ball provides a measure of anisotropy *α* that is used to weight the influence of the *q*-ball on the particle trajectories: particle inertia increases for low anisotropy *q*-balls.

The behavior of the particle speed direction can be understood from a simple mechanical analogy: at each step of the trajectory sampling, the new speed $\overset{\to }{v(i+\delta t)}$ results from a tradeoff between inertia ($\overset{\to }{v(i)}$) and a force stemming from the local *q* ($\overset{\to }{v_{q}}$) (see Figure 2):

(2) $$\overset{\to }{v(i+\delta t)}=\alpha \overset{\to }{v_{q}}+(1-\alpha )\overset{\to }{v(i)},$$

where *α* is a parameter ranging between 0 and 1 that will be described latter. The orientation $\overset{\to }{v_{q}}$ of the force acting on the particle is chosen randomly inside a half cone defined from the inertia direction $\overset{\to }{v(i)}$. The probability distribution $F_{q}(\overset{\to }{v(i)})$ driving this drawing corresponds to the restriction of the *q* to this half cone. This distribution, called further the restricted fiber ODF, is build after renormalizing the *q*-ball values within the half cone. The renormalization and the drawing are performed among the sampled directions of the *q*-ball belonging to the half cone. Therefore, the maximum of the *q*-ball inside the half cone has the highest probability to influence the particles.

The weight *α* is the standard deviation of the *q*-ball normalized by its maximum in the field, computed after removing the 5% highest values to prevent the influence of spurious extreme values resulting for instance from motion artifacts. Hence, this weight depends on the location in the *q*-ball field. In fact, *α* is a measure of anisotropy [54]. A slice of *α* can be visualized in Figure 7(b) For isotropic voxels, *α* parameter is small and the algorithm favors inertia direction; while for anisotropic voxels, *α* parameter is large and the algorithm favors *q*-ball distribution (see Figure 2). Hence, the particles have a tendency to proceed further in the initial direction in voxels where the diffusion peaks are not reliable.

The particle trajectory regularization depends on three parameters:

- the half-cone angle is used to discard the diffusion peaks leading to high curvature of the trajectory;
- the *q*-ball standard deviation (*α* parameter) tunes the weight of the inertia;
- the constant sampling *δt* provides another level of tuning: increasing the trajectory sampling decreases curvature regularization.

In this paper, the influence of these ad hoc parameters is not explored. In the following, we use a half-cone angle of 30 degree and a constant sampling equal to half the minimal voxel size, namely 1 mm.

#### 2.2.2. Validation on a fiber crossing phantom

The lack of knowledge about the white matter organization of the human brain is a huge handicap for the community developing fiber tracking algorithms. Considering the complexity of the MR diffusion signal, it is rather difficult to validate such algorithms using only simulated data. Therefore, the development of phantoms with known geometry is in our opinion crucial for a better understanding of the algorithm behaviors [51].

**Figure 3:**
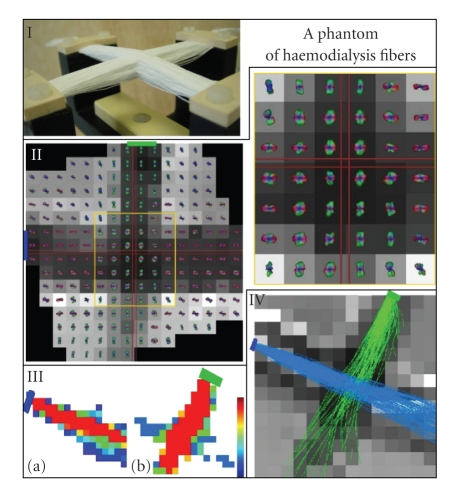
I: a phantom of fiber crossing. II: a slice of the 512 directions *q*-ball acquisition with a zoom on the crossing area. *q*-balls are superimposed on a T2-weighted MR image whose intensity is related to water amount. *q*-balls and MR data have been slightly rotated in order to simplify the reading of the *q*-ball 3D color code. Green and blue rectangles denote the regions of interest at the origin of fiber tracking. III: slices of the number of particles crossing each voxel at the end of the fiber sampling (left: blue bundle, right: green bundle). IV: trajectories selected by a threshold on the particle density map for each bundle. A T2-weighted slice of the phantom crossing the bundles is used as a background and hides some trajectories.

For this purpose, we have designed a phantom corresponding to two intersecting fiber bundles. It consists of sheets of parallel haemodialysis fibers (Gambro, Polyflux 210 H) with an inner diameter of 200 micrometers and an outer diameter of 250 micrometers. Sheets of two different orientations intersecting at 90 degrees were stacked on each other in an interleaved fashion [51]. Crossing thickness is above 2 cm. Fibers are suspended and hold by two arms as seen in Figure 3. Fibers are permeable to water. They are dived in pure water mixed with gadolinium.

We performed DW-MRI acquisitions on a 1.5 Tesla Signa Excite II MRI system (GE Healthcare, Milwaukee) with maximal gradient intensity of 40 mT m^-1^. Acquisitions were performed with spin-echo EPI sequence and Stejskal and Tanner diffusion gradient [55]: *b* value is 700 s/mm^-2^, equivalent to 2000 s/mm^-2^ for diffusion in brain white matter, 512 orientations of the diffusion gradient (HARDI), matrix 64 × 64, in-plane voxel resolution 3.75 × 3.75 mm, slice thickness 2.0 mm, TE 66.6 milliseconds, TR 3000 milliseconds, 1 shot, field of view 24 cm. Spatial distortions of the diffusion-weighted images induced by eddy currents were corrected before estimating the *q*-ball field. This correction relies on a slice-by-slice affine geometric model and maximization of mutual information with the diffusion free T2-weighted image [56].

A slice of the *q*-ball field is shown in Figure 3. Unfortunately, because of a difficult positioning of the phantom due to the shape of its container, the two crossing bundles are not parallel to the slice axes. To clarify the visualization of the *q*-ball data based on color encoding, a rotation around the*z*-axis has been applied to the data before visualization. Then the orientation of each bundle corresponds to a pure color in the *q*-ball meshes (green and red). A zoom on the crossing area highlights the additional information provided by the *q*-ball compared to a tensor model. The diffusion peaks, however, would provide a better angular discrimination with higher *b* value (*q*′).

For each bundle, the tracking algorithm is fed with a ROI made up of 3 voxels, using 3 × 130 particles. The particles propagate throughout a mask defined from the T2-weighted image. This mask corresponds to the part of the field of view including the artificial fibers. It was defined from a high threshold on intensity (the voxels including fibers contain less water, which leads to less signal), followed by a morphological closing in order to fill up spurious holes. A slice of the two resulting particle density maps is shown in Figure 3. A threshold of 5 particles is applied to these maps in order to create a mask used to select reliable trajectories. The remaining trajectories do not include any spurious fork in the crossing area.

**Figure 4:**
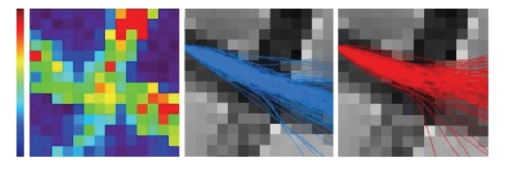
(a) A slice of the normalized standard deviation of the *q*-ball (*α*). (b) Particle trajectories in the initial *q*-ball field (T2-weighted image behind). (c) Particle trajectories in the field where the *q*-balls of the crossing area have been rotated around the *z*-axis (20 degrees). A lot of trajectories are bended which shows that the algorithm is not overregularized.

A second experiment was performed to check that the successful result was not only due to the fact that the phantom bundles have a straight geometry. With such a geometry, indeed, curvature regularization is sufficient for the particles to pass through the crossing area without trouble. For this second experiment, a 20 degree rotation around the *z*-axis was applied to the *q*-balls of the crossing area corresponding to the zoom of Figure 3. Then the tracking algorithm was triggered with the same set of particles as for the first experiment using first the initial *q*-ball field and second the modified field. However, the particles could propagate throughout the whole field (no mask) and no filtering of the trajectories was applied using the particle density map. The results shown in Figure 4 prove that the curvature regularization does not prevent the particle to follow the rotated fiber direction indicated by the *q*-balls of the crossing area. This observation means that the *q*-balls of the crossing area are anisotropic enough to oppose the particle inertia.

#### 2.2.3. The mask of propagation

The particles propagate throughout a mask and trajectories stop only when they leave this mask. Thanks to the perfect alignment between the high resolution T1-weighted image and the diffusion dataset, a first refinement of our initial method could be designed: a mask preventing the particle from crossing the cortical folds. This can be achieved using a pipeline of processing dedicated to T1-weighted images and proposed by BrainVISA. A mask of the brain is first defined through bias correction, histogram analysis, and mathematical morphology [57, 58]. Then an homotopic chain of processing is providing a hole-proof skeleton of the cerebrospinal fluid that can be viewed as a negative mold of the brain filling up the folds [41]. A dilation is applied to this skeleton through the addition of its 26-connected neighbors in order to create a wall that particles cannot cross whatever their trajectory. The dilated skeleton is finally removed from the brain mask in order to yield the mask of propagation. A slice of this mask is proposed in Figure 7(a). Because of the 1 mm resolution of T1-weighted images and the minimal 2 mm thickness of the cortex on both sides of the folds, this dilated sleketon does not include any white matter voxel. In fact the mask is made up of the white matter and of a thin layer of cortical grey matter.

#### 2.2.4. Sampling of long fascicles

Let us consider a long fascicle of 5 cm. Let us consider also a particle traveling step by step along this fascicle with 1 mm jumps. Let us assume that the *q*-balls located along this fascicle always present a very strong peak in the direction of the fascicle. This means that at each step, the sampling of the restricted fiber ODF $F_{q}(\overset{\to }{v(i)})$ providing the next direction to follow has a very high chance to select the actual fascicle direction. However, for the particle to reach the end of the fascicle, this event has to occur 50 times in a row, which is almost impossible except with *q*-ball close to Dirac distributions. Therefore, even with a very large number of particles, our probabilistic tractography is highly biased toward short range connections. Two refinements of our sampling strategy have been introduced to improve the situation:

- a processus creating child particles while sampling long fascicles supported by high probabilities when drawing from the restricted fiber ODFs;
- a sharpening of the restricted fiber ODFs: the drawing weight is concentrated around the maximum of the *q*-ball in the underlying half cone.

**Figure 5:**
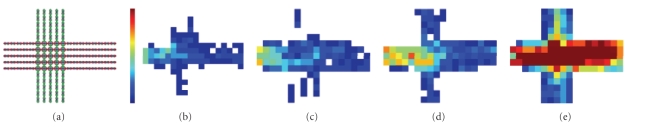
Tuning the child particle creation process. Tracking is performed from a R0I of 5 × 3 voxels located at the left extremity of one of the bundles of the simulated crossing with 4 particles per voxel and different tunings. For each experiment, a density map is computed: each voxel reports the number of times it has been intersected by a trajectory. For one slice of the phantom: (a) *q*-ball, (b) no child particles. For other experiments, each initial particle fathers a child particle at each step as long as the restricted ODF probability remains above a percentage of the local maximum. (c) Birth threshold of 97%, (d) birth threshold of 95%, (e) birth threshold of 90%.

The creation of child particles follows an intuitive heuristics, tuned by a threshold on the probability drawed from the restricted fiber ODFs. At each step, the threshold is defined as a percentage of the maximum of $F_{q}(\overset{\to }{v(i)})$. A mother particle fathers a child at each jump as long as the series of probabilities drawed since the beginning of its trajectory remains above the threshold. The mother particle becomes sterile after the first drawing under the threshold. The children are not fertile. Their initial speed is the same as the mother's one. The process is illustrated in Figure 5 using artificial *q*-ball fields computed from a random walk simulator briefly described in the appendix [59]. From these simulated crossing bundles, we perform tracking from a R0I located at the left extremity of one of the bundles using different thresholds. For each experiment, a density map is computed: each voxel reports the number of times it has been intersected by a trajectory. Without child birth, the density drops down rapidly with the distance from the initial ROI: most of the particles rapidly quit the bundle. With the child creation process, it is possible to find a threshold compensating the particle lost.

#### 2.2.5. Sharpening the *q*-ball

**Figure 6:**
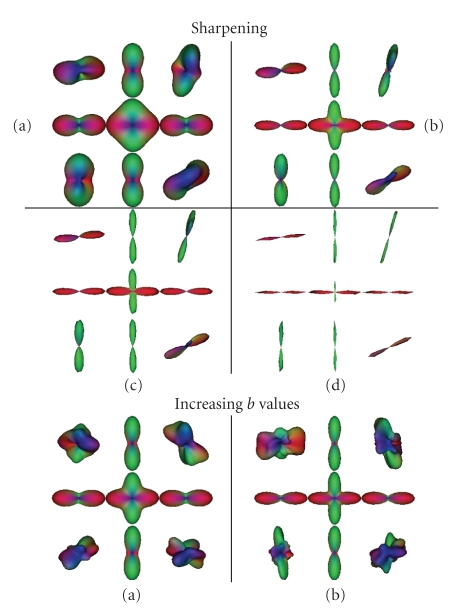
Influence of the sharpening parameter on *q*-balls. Simulated crossing (90 degrees). *Top:* (a) native meshes, *b* = 700 s/mm^−2^. (b) Sharpened meshes with *S* = 0.2. (c) Sharpened meshes with *S* = 0.1. (d) Sharpened meshes with *S* = 0.002. *Bottom:* simulated data with high *b* values. (a) Native meshes *b* = 2000 s/mm^−2^. (b) Native meshes, *b* = 4000 s/mm^−2^.

Working with *q*-ball fields raises the issue of the optimal *b* value for *q*-ball acquisitions. Increasing the *b* value, indeed, sharpens the Bessel kernel and increases the ability to resolve distinct diffusion peaks but at the cost of a lower signal-to-noise ratio. The 3000 s/mm^2^ *b* value used in this paper leads to a very low signal-to-noise with our scanner, that is fortunately compensated by the 200 directions of diffusion sensitization. The resulting *q*-balls are not focused enough around the putative fiber direction to be used safely as fiber ODF. Therefore, we propose to sharpen the *q*-ball restriction in inertia half cones in order to build the restricted fiber ODF. For this purpose, $F_{q}(\overset{\to }{v(i)})$ is defined further as

(3) $$F_{q}(\overset{\to }{v(i)})(d)=\frac{1}{N}\text{exp}(\frac{1}{S}\frac{\psi (d)-\psi_{\text{min}}}{\psi_{\text{max}}-\psi_{\text{min}}}),$$

where *S* is the sharpening parameter, *ψ*_max_ and *ψ*_min_ are the extrema of the *q*-balls in the inertia half cone, and *N* is a normalizing factor. As *S* tends to 0, the restricted ODF gets closer to a Dirac function putting most of the sampling weight around the local maximum of the *q*-ball supposed to indicate the most reliable trajectories. An illustration of the effect of sharpening applied to the *q*-balls is proposed in Figure 6 thanks to the diffusion simulator described in Appendix. Simulated data with a weighting in diffusion of *b* = 700 s/mm^−2^, can be compared to simulated data with higher *b* value. Decreasing the sharpening parameter, as well as increasing the *b* value, tends to refine the *q*-balls to their maxima. It should be noted that our naive sharpening approach will have to be improved, because it is not robust to large differences in the amplitudes of the peaks of the *q*-ball ODF. A very attractive candidate based on spherical deconvolution has been recently proposed by Descoteaux et al. [23].

**Figure 7:**
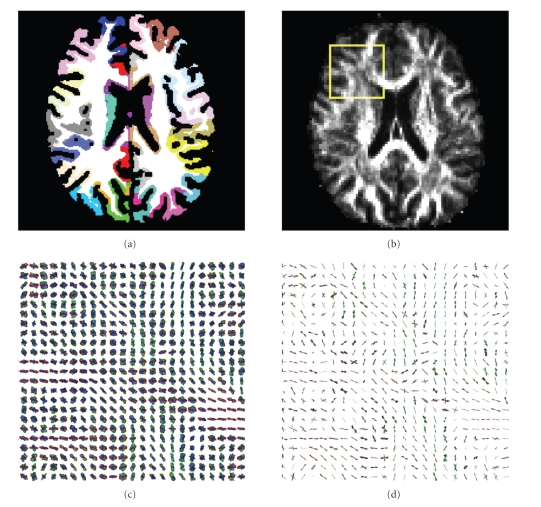
Propagation mask and tuning of inertia. (a) A slice of the propagation mask superimposed with the gyral parcellation. (b) Alpha map: alpha is a measure of anisotropy increasing the influence of the *q*-ball versus the particle inertia. (c) Zoom on the *q*-ball of the yellow area. (d) Zoom on the *q*-ball of the yellow area with a global sharpening focusing the distribution weight around the *q*-ball maximum *S* = 0.1 (it should be noted that during the tracking, the sharpening is performed locally inside a half cone of directions defined by the particle inertia).

**Figure 8:**
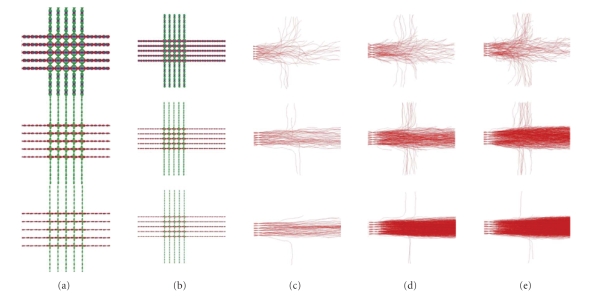
Influence of sharpening parameter on *q*-ball meshes and tracking. Top: no sharpening. Middle: *S* = 0.2. Bottom: *S* = 0.1. (a) *q*-ball zoom on crossing, (b) slice of *q*-ball simulated data, (c) no child fibers, (d) threshold birth of 3%, (e) threshold birth of 5%.

Figure 7 shows also the global sharpening of the *q*-ball on our human diffusion data. It should be noted that our sharpening approach preserves the shape of the *q*-ball around a local maximum. For instance, a fan of fibers should lead to a crest of high probability, therefore preserving more of the information provided by the fiber ODF than the simple selection of the local maximum. The tuning of the sharpening parameter mixed up with the child creation process is illustrated with simulated data (cf. Figure 8).

In conclusion, this set of refinements improve the behavior of our framework relative to long fibers. In the following, the child creation threshold and the sharpening parameters have been set following our experiment with simulated phantoms. We are well aware, however, that more careful studies have to be carried on for a better tuning of these parameters in noisy situations. Nevertheless, it should be noted that the most noisy *q*-balls occur inside grey-matter, where anisotropy is low. Therefore, they should not be able to stand strongly against the particle inertia. It should be noted that the *q*-ball field visualizations provided in this paper do not always scale the *q*-ball according to anisotropy (cf. Figure 1). Hence, the grey matter *q*-balls visibility is sometimes higher than their influence on the tracking process.

### 2.3. Connectivity-based parcellation

The tractography method introduced above is used to compute the connectivity profiles of all the voxels of the cortical mantle. Similarities between these profiles are used to parcellate the cortex into areas with stable profiles. The parcellation is computed in two steps. The cortex is first parcellated into large gyri, then each gyrus is parcellated into smaller entities according to the profiles of connectivity to the gyral parcellation.

Projecting a parcellation from one anatomical structure toward another using tractography-based connectivity is bound to become a very powerful tool for neuroscience. Its potential has been shown for instance to project a cortical lobar parcellation toward the thalamus [31] or toward the corpus callosum [60]. A very attractive extension of the same idea will consist in projecting areas mapped with functional imaging. Another variant of the idea described in this paper is the “feedback” projection: our clustering aims at parcellating further the initial parcels.

For the two pioneer applications mentioned above, the projection is performed from the parcel with the maximum connectivity strength. In this paper, this “maximum connectivity-strength-” type of projection is used to initialize a non supervised clustering algorithm with a more ambitious objective: the clustering is performed according to similarities between vectors made up of the connectivity-strength with each of the parcels. This objective is mandatory to address the cortex parcellation, because what defines a cortical area is not one but a set of connections [27].

#### 2.3.1. Gyral parcellation

**Figure 9:**
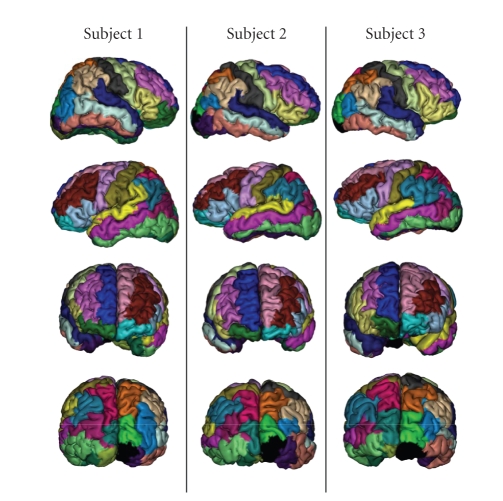
External views of the gyral parcellation of the two hemispheres of the three subjects. The color code is used in most of the following figures about connectivity-based parcellation.

The gyral parcellation used by our method has been computed from the T1-weighted image, using pipelines of processing embedded in BrainVISA framework. The cortical folds are first extracted one by one [61], then a pattern-recognition system made up of 500 multilayer perceptrons gathers the elementary folds to identify the main sulci [62]. For the three brains of this paper, a human expert checked the result of this recognition and performed some manual corrections. Finally, a last pipeline of processing uses the sulci to perform an automatic parcellation of the cortical surface into gyri [35]. This parcellation of the cortical surface is projected to the whole cortical mantle using standard techniques of mathematical morphology. A set of views describing the gyral parcellations obtained for the three brains is presented in Figure 9. The result is relatively stable across subjects and hemispheres, although the large variability of the folding patterns and some weaknesses of our system lead to some differences. The color code labelling the 36 gyri is used further in the rest of the paper to describe the projected parcellations.

#### 2.3.2. Connectivity profiles

The parameters of the tractography are the following: the threshold controlling the child creation is 5% and the sharpening parameter is 0.1. For practical reasons, only four mother particles are triggered from each voxel of the cortical mantle defined at the resolution of the T1-weighted image. For a given voxel, the four mother particles father an average of 80 children, and a maximum of 250 children. The number of voxels making up a gyrus is variable: 25000 voxels for a typical superior parietal gyrus and 45000 for a typical superior frontal gyrus. For practical reasons, the tractography was performed for only 14 of the gyri, covering the external part of frontal and parietal lobes in each hemisphere. The whole computation took four days on a network of 14 computers. For each voxel, we count the number of particles reaching each of the 36 gyri, including mothers and children, in order to build the connectivity profiles. The number corresponding to the gyrus including the voxel is set to zero to discard suspicious particles propagating mainly throughout grey matter.

**Figure 10:**
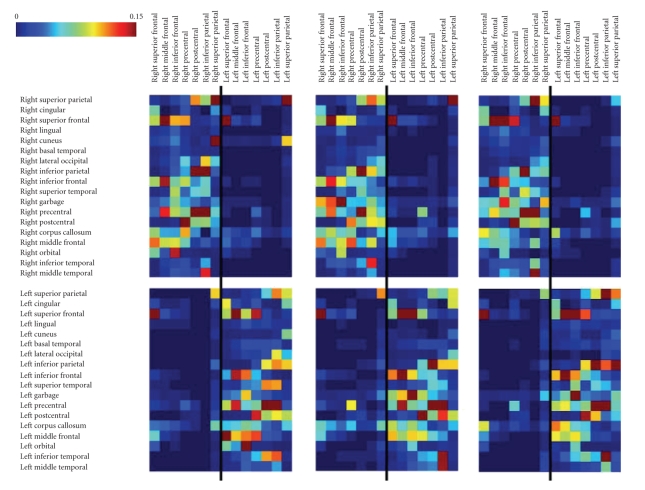
Global connectivity matrix of the gyri implied in the tractography (subjects 1, 2, and 3).

Considering a gyrus of *n* voxels, the whole information is gathered into a *p* × *n* connectivity matrix *C*. The columns of the matrix are normalized for the number of particles in order to get comparable profiles. Hence, a connectivity profile looks like a probability distribution. It is fairly impossible to visualize globally the matrix obtained for one gyrus. We observed that a lot of voxels are connected to more than one gyrus, which justifies the idea of basing the clustering on the connectivity profiles. To provide a global overview of the tractography, each matrix was averaged to a mean column vector. Gathering the resulting vectors leads to the connectivity matrix of the gyri. The matrices obtained for the three subjects are proposed in Figure 10. They present a lot of similarities across subjects and hemispheres.

A careful observation leads to discovering a specificity of this matrix: the symmetric connectivity strengths estimated for a pair of gyri, when they are computed, are not necessarily identical. In fact, this is not necessarily a failure of the system. Indeed, these two strengths should be identical only if the connections between both gyri are symmetrical.

#### 2.3.3. Clustering

**Figure 11:**
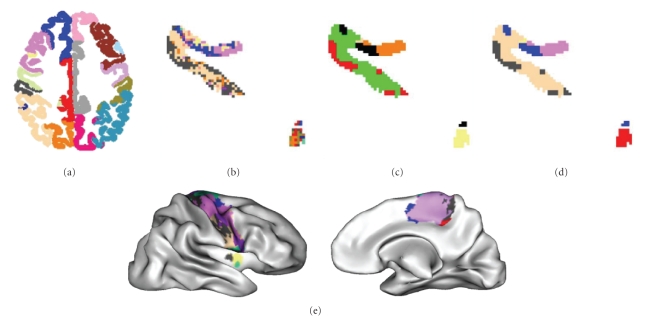
Slice results in a precentral right gyrus. (a) Slice location. Precentral gyrus is in light green color, (b) max of connectivity results for the same slice, (c) clustering results for the same slice, (d) clustering result for the same slice with the labelization of the most connected gyrus. Light pink: inferior parietal gyrus, yellow: inferior frontal right gyrus, blue: superior frontal right gyrus, purple: medial frontal, gray: postcentral right gyrus. (e) 3D projection of the clustering with labeling of the most connected gyrus.

The nonsupervised clustering approach used to gather voxels with similar profiles is based on the classical *K*-means algorithm associated with a spatial regularization provided by a Markov random field model [63]. In order to stabilize the *K*-means approach, which is known for its high dependence on initialization, we use the maximum connectivity strength idea mentioned above [31, 60]. Hence, the gyrus is first split in potentially 35 areas, each voxel being associated with the gyrus corresponding to the highest value in its profile. Usually, the actual number of areas is much lower, which can be understood considering the average profile of each gyrus (see Figure 10). The resulting parcellation is especially noisy, which can be visualized in the example provided in Figure 11(b). This observation calls for several possible explanations.

1. The poor sampling of white matter performed during the tractography (only four mother particles) could lead to a weak estimation of the maximum strength.
2. Many voxels turn out to have several important connections of equivalent strength; hence, in a group of nearby voxels with similar profiles, the winner gyrus could switch randomly between the competing gyri.
3. The initial tracking directions provided by the *q*-balls of cortical grey matter could often be spurious, either because of noise or because of some microstructures of the cortical layers corresponding to tangential myelinated fibers.

These difficulties would benefit a lot from the addition of contextual knowledge in the clustering process, which is done further using a simple model of spatial regularization stemming from the domain of Markov random fields. Furthermore, the second difficulty could be largely overcome by the use of the complete connectivity profiles during the clustering, which is done further by the *K*-means algorithm. The *K* largest areas of the maximum connectivity parcellation are used to compute the *K* initial centroids of the *K*-means.

 Let us note *Y* = (*y*_1_,…,*y_n_*) the observable set corresponding to the data: the normalized connectivity profiles of the *n* voxels of the gyrus. Let us note *X* = (*x*_1_,…,*x_n_*) the unobservable (hidden) set corresponding to the cluster assignments of the voxels. Each hidden variable *x_i_* denotes the cluster label of the point *i* and takes values from the set of cluster indices (1,…,*K*). Finally, let *M* = (*μ*_1_,…,*μ_K_*) denote the cluster centroids. When considering the conditional probability *p*(⋅ | *x* = *h*), the probability of generating a profile from the hth cluster, we restrict our attention to probability densities from the exponential family, where the expectation parameter corresponding to the hth cluster is *μ_h_*. Using this assumption and the bijection between regular exponential distributions and regular Bregman divergences [64], the conditional density for observed data, which corresponds to data attachment, can be represented as

(4) $$p(y_{s}|x_{s})\alpha \,\;\;\text{exp}\,\;\;(-D(y_{s},\mu_{s})).$$

In the following, *D* is the Euclidean distance, but it would be of interest to compare with results obtained using KL-divergence.

The Markovian prior probability of our clustering method is the standard Potts model, which penalizes the number of adjacencies between clusters. This is minimizing the area of each cluster interface hence leading to spatially smooth clusters. This probability is a Gibbs distribution based on potentials acting on the set of cliques of order 2 called *C*_2_:

(5) $$\begin{array}{ll} U_{2}(x_{r},x_{s}) & =-\beta \;\,\text{if}\;\;x_{r}=x_{s},\\ U_{2}(x_{r},x_{s}) & =+\beta \,\;\text{if}\,\;\;x_{r}\,\;\neq \;x_{s}. \end{array}$$

In the following *β* is fixed to 0.05.

The a posteriori energy whose minimum is the target of the clustering is finally

(6) $$U(x|y)=\underset{s\in [1,n]}{\sum }D(y_{s},\mu_{s})+\underset{\left(r,s)\in [1,n\right]}{\sum }U_{2}(x_{r},x_{s}).$$

This energy is minimized alternating the standard ICM algorithm and the centroid update. The initial centroids are the average profiles of the *K* largest areas of the max-connectivity parcellation.

- Initialize the *K* clusters centroids *M* = (*μ*_1_,…,*μ_K_*).
- Repeat until convergence.
  - Given centroids *M* = (*μ*_1_,…,*μ_K_*), reassign cluster labels to minimize U using ICM.
  - Given cluster labels, recalculate centroids *M*.

Voxels with no starting particles or whose particles end their trajectory in the starting gyrus have no data attachment and do not contribute to centroid computation. For the sake of simplicity, for all the results presented in this paper, each gyrus has been split using a *K*-means with ten clusters. The spatial regularization term sometimes reduces the final number of clusters. The fact that a frequent profile including more than one strong connection may be split into two clusters during initialization and merged back during the *K*-means can also reduce the final number of clusters. But we have no reason to expect that our procedure systematically performs a successful split and merge, which will be discussed further later on. Exploring techniques to adapt the number of initial clusters to each of the gyrus is difficult and beyond the scope of the paper. Number ten was chosen because it was larger than the number of significant parcels obtained by the max-connectivity clustering for most of the gyri. An additional motivation was the fact that standard architectonic parcellations do not split the gyri of our macroscopic parcellation into more than ten areas. The denoising of the max-connectivity parcellation obtained by our nonsupervised regularized clustering is illustrated in Figure 11.

## 3. RESULTS

### 3.1. Color coding the parcellation

In the following, we illustrate the method developed in this paper through a study of the reproducibility of the parcellation across three subjects. The seven gyri parcellated in each hemisphere are the three elongated gyri of the external part of the frontal lobe, called superior, medial, and inferior (F3, F2, and F1 in monkey literature), the precentral (motor) and postcentral (somesthesic) gyri, the superior and inferior parts of the external parietal lobes. The results of such a 3D parcellation are especially difficult to visualize or compare. To simplify the comparison across subjects, we have decided to label each cluster with the color of the most connected gyrus, using the color code introduced in Figure 9. This color code has some limitations: some cluster boundaries are hidden, either because the stereotype profile of two neighboring clusters share the same maximum, or because two neighboring clusters belong to two different gyri.

### 3.2. 3D projections

**Figure 12:**
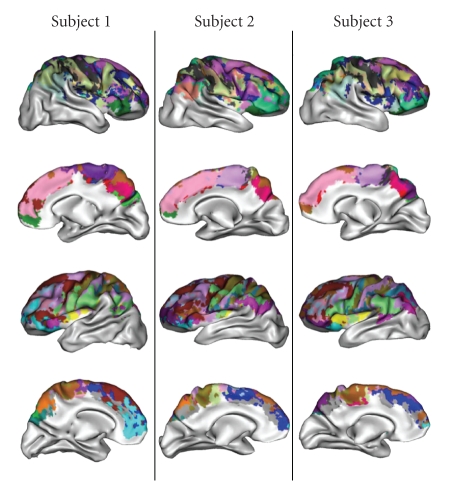
Projection of the parcellation with cluster colors corresponding to the most connected gyrus.

The second choice has been to develop a method to present the results in 3D. For this purpose, a spherical mesh representing the grey/white interface is computed using BrainVISA [41]. This mesh is slightly inflated in order to preserve only the largest folds corresponding to the main boundaries between the macroscopic gyri. Then the node of the mesh is colored with the label of the closest parcellation voxel. This projection provides an interesting glimpse of the global parcellation but hides a lot of information when several clusters compete to color a mesh node. This method has been applied to the three subjects to build the Figure 12. The similarities observed across subjects are relatively encouraging, but some severe differences are observed for some clusters. We will see further that these differences result from weaknesses of our color coding, when a stereotype profile includes two equivalent strong connections.

### 3.3. Spatial normalization

**Figure 13:**
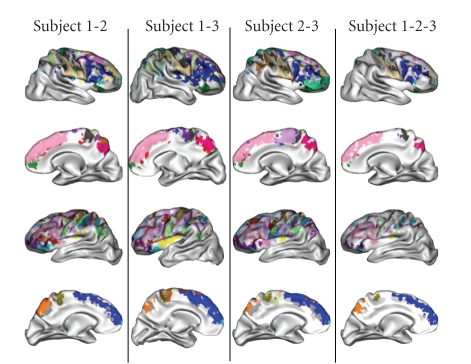
Intersection of the color-coded parcellations after affine spatial normalization.

While the method described in this paper aims at developing structural representations of the cortex supposed to overcome some weaknesses of the standard spatial normalization framework, we have tried to support the comparison of subjects using brain alignment. Affine transformation aligning the T1-weighted image of each of the subjects toward a corresponding template was computed using SPM2 [65]. These transformations were applied to the parcellations of the cortical mantles using a nearest neighbor interpolator. Finally, for each pair of subjects and for the triplet of subjects, the intersections of the parcellations were computed. The result of such an intersection includes only voxels with the same colors in the compared parcellations. Each of these intersections are projected on one of the compared brains in Figure 13. This figure highlights a lot of similarities when comparing 2 subjects, and a sharp decline with three subjects (cf. Figure 13). This decline is partly due to nonperfect spatial normalization, but also largely to the color-code problem already mentioned above.

To help the decoding of the anatomical information embedded in our color code, we collected the list of connections surviving after the intersection of the three subjects.

**Frontal projections**

Frontal superior gyri project in inferior frontal, in precentral, in orbital frontal, and in cingular gyri of the same hemisphere and in frontal superior gyrus of the other hemisphere via corpus callosum. Furthermore, right superior frontal gyrus projects in right middle frontal gyrus. Medial frontal gyri project in inferior and superior frontal gyri and in precentral gyrus of the same hemisphere. Inferior frontal gyri project toward superior frontal and orbital frontal gyri of the same hemisphere. Right inferior frontal gyrus has additional connections in right lingual and right superior temporal gyrus. Left inferior frontal gyrus project in precentral left gyrus.

**Precentral projections**

Precentral gyri project in superior and medial frontal and in postcentral gyri. Right precentral gyrus has additional connections in right inferior frontal, right inferior parietal, and right precentral gyri. Left precentral gyrus projects in left inferior temporal gyrus and in right precentral gyrus via the corpus callosum.

**Postcentral projections**

Postcentral gyrus projects toward inferior parietal, superior temporal, and precentral gyri of the same hemisphere. Right postcentral gyrus projects in right inferior frontal, left postcentral gyrus projects in left superior parietal gyrus.

**Parietal projections**

Right superior parietal gyrus projects in occipital lateral and precentral gyri of the same hemisphere and in left superior parietal gyrus via corpus callosum. Left superior parietal gyrus connects cingular, postcentral and inferior parietal of the same hemisphere and right superior parietal via corpus callosum. Right and left inferior parietal gyri have main connections in superior parietal and precentral gyri of the same hemisphere. Furthermore, right superior parietal gyrus has connections in occipital lateral and middle temporal gyri of the right hemisphere; left inferior parietal gyrus has also connections in left inferior and superior temporal gyri.

### 3.4. Matching clusters across subjects

**Figure 14:**
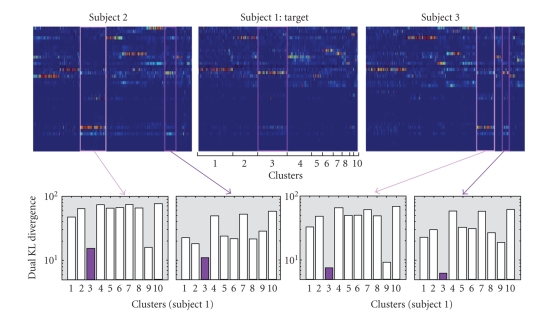
Matching clusters of the precentral gyrus across subjects using distance between mean connectivity profiles provided by dual KL divergence (see text).

We mentioned already that the simple-color coding proposed above to compare the parcellations has some flaws with profiles including several strong connections. We will now illustrate this aspect with two examples. Let us first consider the top of the right precentral gyrus in the internal face presented in the second row of Figure 12. We observe a large dark violet cluster for subject 1. For the two other subjects, however, we observe a large light violet cluster, surrounded by a few dark violet points. To understand this configuration, we computed the matrix of profiles of the right precentral gyrus for each of the subjects (cf. Figure 14). Since this gyrus is made up of more than 30000 voxels, the snapshots expose only a few profiles picked up randomly. The profiles are ranked according to the result of the clustering. The ten clusters of subject 1 are underlined. The five dark and light violet clusters mentioned above are highlighted in the profile matrices. It can be observed that the profiles of these five clusters are very similar, presenting several strong connections with the same gyri. The most connected gyrus, however, is not the same for each of the subjects. This observation can be confirmed quantitatively by comparing the average profiles across subject using the dual KL divergence, a measure of distance between probability distributions. The charts provide the distances of the violet clusters of subjects 2 and 3 to each cluster of subject 1. The dark violet cluster of subject 1 turns out to be each time the closest one, which shows that the five clusters should be matched as corresponding to the same anatomical entity. The main connections of this entity are the gyrus located above corpus callosum, the left postcentral gyrus and the left superior frontal gyrus.

## 4. DISCUSSION AND CONCLUSION

The method exposed in this paper is still largely exploratory, relying on several parameters whose influence should be studied. However, the new opportunities for neuroscience provided by the connectivity-based parcellation paradigm are very attractive [31, 32, 34, 60] and we need to push the exploration as far as possible before tuning the method. It is too early to decide if the connectivity matrices and the parcellations inferred from our framework are meaningful, but their level of reproducibility across subjects is impressive. It should be noted that according to anatomical knowledge, architectonic areas can double or triple in size from one subject to another [28]. Therefore, there is no simple way to quantify the reproducibility of our parcellations. The mandatory approach will be a correlation of such connectivity-based parcellations with mappings obtained from functional imaging or postmortem anatomical studies [27, 32].

In our opinion, despite our care to improve the sampling of long bundles, an important weakness of our framework is the bias of our probabilistic tractography for the short tracts, which could explain the small amount of inter-hemispheric connections. This bias stems from the way we introduced the trajectory regularization in our framework, requiring the particles to follow the fascicles from the beginning to the end. Some local regularization could be designed to overcome the problem, extending the method initially introduced by Behrens et al. [12], or using the spin glass framework introduced by our group [10, 15]. Another solution could be provided by the normalization of the connectivity strength relative to the path length [34] or using more sophisticated models of the length dependence [66].

An alternative to the probabilistic framework for the computation of the connectivity profiles lies in the methods based on front propagation assimilating the tracts to geodesics [67–69]. While these methods seem prohibitively expensive for the computation of the one million connectivity profiles used in this paper, they do not suffer from the sampling weaknesses of our particle-based approach. Furthermore, the sampling used in this paper for cortical mantle is unrealistic relative to the spatial resolution of the diffusion data. Therefore, dealing with a more reasonable sampling of the cortex is one of the key future refinements of our method. Considering that the current spatial resolution of diffusion data cannot give access to the myeloarchitecture of the cortical layers, an attractive solution would be to address the parcellation of a spherical model of the cortical surface [28], following the approach proposed in [70] to align connectivity matrices. The surface-based approach would largely reduce the number of connectivity profiles to be clustered. Surface-based analysis would overcome the piling up of different clusters orthogonally to the cortical surface. A surface-based 2D model for Markovian regularization would be more reliable than our 3D approach depending on the sampling of the cortical mantle in the direction orthogonal to the surface. The initial starting speed could be defined by the surface normal, and the visualization of the parcellation would be straightforward. Finally, this approach would fit the current knowledge of the columnar organization of the cortex: the large scale connectivity is shared by small groups of neurons organized orthogonally to the cortical surface [27, 71].

**Figure 15:**
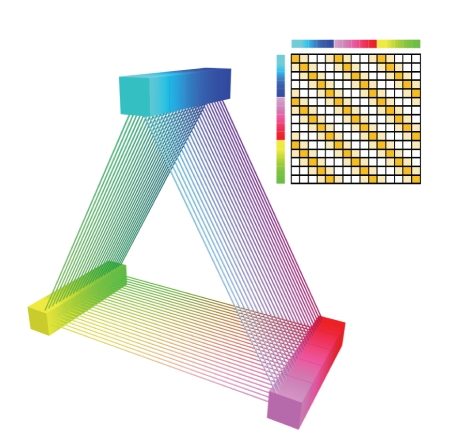
A synthetic retinotopic-like network of three areas and its connectivity matrix. A clustering based on the profiles of connectivity with the whole brain will gather voxels with similar retinotopic coordinates (light colors with light colors, dark colors with dark colors). In return, if the three retinotopic areas belong to different parcels of a large scale parcellation, a clustering based on the profiles of connectivity with this parcellation will split the system into the three areas.

Our framework for clustering requires an input parcellation whose influence on the result can be discussed a lot. However, whatever the weaknesses of the input parcellation, we would like to advocate that basing the clustering on similarities between profiles of connectivity with a parcellation rather than the whole brain, like in the work of the Oxford group mentioned in the introduction, can lead to two very different almost orthogonal results [32]. This is illustrated by Figure 15, which depicts a synthetic system of three areas linked by a retinotopic-like network of connections. A clustering based on the profiles of connectivity with the whole brain will gather voxels with similar retinotopic coordinates. In return, if the three retinotopic areas belong to different parcels of the initial large scale parcellation, a clustering based on the profiles of connectivity with this parcellation will split the system into the three areas (areas blue, yellow, and red). This example illustrates the richness of the world of possibilities offered by the connectivity-based parcellation paradigm.

Following the previous discussion, the dependence of the output parcellation on the input parcellation, provided that this one can be defined in a reproducible way across subjects, is a richness rather than a problem. It should be noted also that the idea of performing a feedback projection from one parcellation to itself can be iterated. This could be interesting to perform some hierarchical parcellation. Adding a merging step between each iteration, in order to gather neighboring clusters with similar profiles, could help to reduce the influence of the initial parcellation. For instance, it could be used to overcome some inadequations of the macroscopic parcellation relatively to the actual architecture. It could also correct some failures of the process defining the gyri occurring for subjects with unusual cortical folding patterns. Finally, this iterative process would turn into a split and merge principle famous in the field of computer vision.

The split and merge approach may be the perfect tool to improve the robustness of the parcellation framework. But the real challenge for the future will be to design a split and merge processus acting on a group of brains, defining the clusters across subjects or matching the individual clusters according to their similarities, as performed above using the dual KL divergence. Group analysis, indeed, seems to be the only way to discard the various bias and artifacts disturbing the tractography and the clustering of the profiles. In our opinion, the usual spatial normalization paradigm will not be sufficient to perform this kind of group analysis: dealing with brain architecture requires a group analysis performed at a structural level [72]. For this purpose, the ideal units relatively to the human brain connectome [27] could correspond to the connectivity-based clusters described in this paper.

## APPENDIX

### Simulated random walk

We have developed a simulation environment limited to tissue made up of tubular fibers and water molecules moving inside and outside fibers [59]. Water molecules follow a Brownian motion and when a particle meets a fiber membrane, it reflects elastically against its surface. This numerical phantom is built from 82 layers of 82 parallel fibers forming two bundles crossing at 90 degrees (diameter 5 *μ*m, interspace 2 *μ*m). The distance between two consecutive random displacements is set to 1 *μ*m with a duration of 82.5 microseconds. Particles are submitted to a Stejskal-Tanner diffusion sensitizations (*δ* = 17.2 milliseconds, Δ = 26.4 milliseconds) with a gradient magnitude of 40 mT/m and a *b* value of 700 s/mm^−2^. With this simulator we get *q*-ball with single fiber population along *x*- and *y*-axes and crossing *q*-ball with both fiber population (3 × 3 × 3 dimension data). We duplicate these data in order to build a 18 × 15 × 5 dataset with a resolution of 1 × 1 × 1 mm^3^.
